# Reducing hypnotic use in insomnia management among Australian veterans: results from repeated national interventions

**DOI:** 10.1186/s12913-018-3443-9

**Published:** 2018-08-09

**Authors:** Lisa M. Kalisch Ellett, Renly Lim, Nicole L. Pratt, Mhairi Kerr, Emmae N. Ramsay, Tammy V. LeBlanc, John D. Barratt, Elizabeth E. Roughead

**Affiliations:** Quality Use of Medicines and Pharmacy Research Centre, School of Pharmacy and Medical Sciences, University of South Australia, GPO Box 2471, Adelaide, South Australia 5001 Australia

**Keywords:** Health service areas, Hypnotics, Insomnia, Trends

## Abstract

**Background:**

The Australian Government Department of Veterans’ Affairs (DVA) Veterans’ Medicines Advice and Therapeutics Education Services (Veterans’ MATES) programme conducted two intervention (March 2009, follow-up intervention June 2012) both of which aimed to reduce hypnotic use among Australian veterans. We evaluated the effectiveness of the interventions, and estimated the associated health consequences.

**Methods:**

Both interventions targeted veterans who had been dispensed hypnotics prior to the intervention. Patient-specific prescriber feedback containing patient details and the volume of hypnotics dispensed, along with tailored educational information, was mailed to general practitioners. Veterans, pharmacists and directors of care in residential aged care facilities were mailed tailored educational information. Interrupted time-series and segmented regression modelling were used to determine the effect of the two interventions on the rate of hypnotics dispensing. The cumulative patient-months of hypnotic treatment avoided as a result of the interventions was calculated. We estimated improvements in health consequences of as a result of hypnotic treatment avoided based on the results of cohort studies in the same population identifying the association between hypnotic and sedative use on the outcomes of falls, and confusion.

**Results:**

After the first Veterans’ MATES intervention in March 2009, hypnotic use declined by 0.2% each month, when compared to the baseline level (*p* = 0.006). The intervention effect was attenuated after one year, and use of hypnotics was found to increase by 0.2% per month after March 2010. Following the second intervention in June 2012, there was a further significant decline in use of 0.18% each month over the 12 months of follow up (*p* = 0.049). The cumulative effect of both interventions resulted in 20,850 fewer patient-months of treatment with hypnotics. This cumulative reduction in hypnotic use was estimated to lead to a minimum of 1 fewer hospital admissions for acute confusion and 7 fewer hospital admissions due to falls.

**Conclusions:**

The Veterans’ MATES insomnia interventions which involved multiple stakeholders were effective in reducing hypnotic use among older Australians. Repetition of key messages led to sustained practice change.

## Background

Insomnia is a common problem affecting one in twenty Australian adults [1], and up to one in ten people aged 65 years and over [1–3]. The recommended first-line treatment for insomnia management is cognitive behavioural therapy [4–6], followed by short term pharmacological treatment using hypnotics if cognitive behavioural therapy is ineffective [5]. Several studies have shown that cognitive behavioural therapy is as effective or more effective than hypnotics in terms of time to sleep onset, amount of time awake during sleep and sleep efficiency, and that the effects of hypnotics are lost following cessation of treatment [7, 8].

Australian guidelines recommend that hypnotics, if required, should only be used short-term (usually no more than 2 weeks), intermittently and at the lowest possible dose [4, 5]. Prolonged use of hypnotics is not recommended as it may lead to tolerance and dependence [4]. Despite this, there is evidence that hypnotics are often used continuously for much longer periods than recommended [9]. In 2006 to 2008, the Australian Bettering the Evaluation and Care of Health (BEACH) survey, which continuously surveyed a rolling cohort of 1000 general practitioners (GPs), reported that 95% of insomnia problems managed at a general practitioner encounter involved a prescription for hypnotics; temazepam (48%) was most commonly prescribed [10]. A US national study from 1996 to 2001 in adults with insomnia or sleeping difficulty reported that hypnotics alone (without cognitive behavioural therapy) were prescribed during half of all patient visits [11].

There is limited evidence on the efficacy of hypnotics for insomnia in the older population [12, 13]. Further, older people are more vulnerable to side effects of hypnotics, with use associated with increased risk of falls and fractures [14–16], confusion and memory impairment [14, 17, 18], motor vehicle accidents [14, 19] and incontinence [20]. In this population, the risks may outweigh any potential benefits [14]. Hypnotic use in Australia remained stable from 2000 to 2007 and decreased marginally from 2007 to 2011 [21, 22]. However, 2006 data showed that hypnotic use while relatively low and comparable for the age groups of 30 to 39, 40 to 49 and 50 to 59, increased from the age of 60 years with peak use in the 95 to 99 year age group [22]. Similarly, the US National Ambulatory Medical Care Survey (NAMCS) data from 1996 to 2001 representing 95 million outpatient visits for insomnia showed that patients aged 65 years and older were 5 times more likely to be prescribed hypnotics compared with those aged 18 to 35 years (OR 4.87, 95% CI 3.64–6.51) [23].

Discontinuation of hypnotics in older people is feasible [9, 24, 25] and has been associated with cognitive and psychomotor improvement [9]. Since most hypnotics are prescribed by general practitioners (GPs) [22], interventions targeting GPs represent an opportunity to reduce prolonged and inappropriate hypnotic use in older people. However, prior research has shown that general practitioners often think patients expect a hypnotic to be prescribed when they visit a GP with sleep problems. [26].

The Veterans’ Medicines Advice and Therapeutics Education Services (Veterans’ MATES) [27] is a national quality improvement program funded by the Australian Government Department of Veterans’ Affairs (DVA) which aims to improve use of medicines and health outcomes for Australian veterans. Almost three quarters of the current veteran population is aged over 65 years (*n* = 143,497, 73%) [28], with associated multimorbidity and multiple medicine use. [29, 30] To date, the Veterans’ MATES programme has run two interventions aiming to improve the use of hypnotics in older veterans. The first intervention was implemented in March 2009 with a follow-up intervention in June 2012, and both aimed to improve the management of insomnia and reduce hypnotic use among veterans in the primary care setting. The messages in both interventions included the need to reduce hypnotic use and to encourage the use of non-pharmacological options to manage insomnia. This study aimed to evaluate the effectiveness of the Veterans’ MATES insomnia interventions in reducing hypnotic use among older Australians, and to estimate health consequences due to changes in hypnotic use following the interventions. The health consequences included changes in hospital admissions due to falls and hospital admissions for acute confusion.

## Methods

**Fig. 1:**
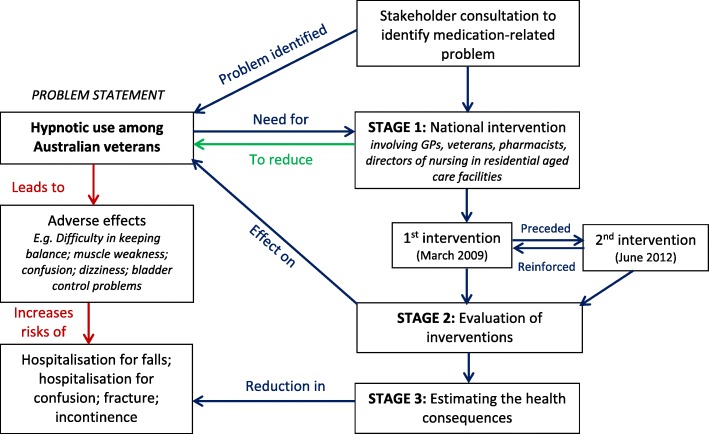
Problem identification and implementation of the Veterans’ MATES interventions to reduce hypnotic use among Australian veterans

The Veterans’ MATES programme, which is underpinned by behavioural theory and strong stakeholder engagement, has been described in detail elsewhere [27]. Specifically, following stakeholder consultation and analysis of health care claims data which identified hypnotic use in elderly as a medication-related problem, a national intervention involving GPs, veterans, pharmacists and directors of care in residential aged care facilities was undertaken to reduce hypnotic use among Australian veterans (Fig. 1). The study was conducted in three stages: (1) implementation of the national interventions; (2) evaluation of the interventions; and (3) estimating the health consequences associated with the interventions.

### Stage 1: Implementation of national interventions

DVA maintains a database of health service claims including hospitalisation admissions, GP visits and pharmaceuticals dispensed which are subsidised for eligible veterans and dependents. Both interventions targeted veterans who were dispensed hypnotics. The hypnotics included benzodiazepine derivatives (Anatomical, Therapeutic and Chemical Classification (ATC) code N05CD), benzodiazepine related drugs (N05CF) or oxazepam (N05BA04). Oxazepam was included as it is often prescribed for insomnia [10].

The first intervention was implemented in March 2009 targeting veterans who had been dispensed at least one hypnotic between 1 January and 31 December 2008. Key messages for the first intervention were to review veterans dispensed hypnotic therapy to reduce use, only use hypnotics short term or intermittently and to cease use where possible. The second intervention was implemented in June 2012 and targeted veterans who had been dispensed at least two hypnotics between 1 October 2011 and 31 January 2012. The second intervention included stronger key messages on ceasing use in longer term users because the first intervention had highlighted that 72% of veterans involved indicated they would be willing to try non-drug options to manage insomnia; and over two-thirds of those using sleeping tablets reported they were willing to reduce the amount they were using [31]. In addition to repeating the key messages from the first intervention, the second intervention also focused on reviewing falls risk as another potential motivating factor for reducing use of the hypnotics; motivating factors are considered a key process within social cognitive theory to support individual behaviour change. Veterans targeted in the first intervention were eligible to be targeted in the second intervention if they were still dispensed a hypnotic on a regular basis.

**Fig. 2:**
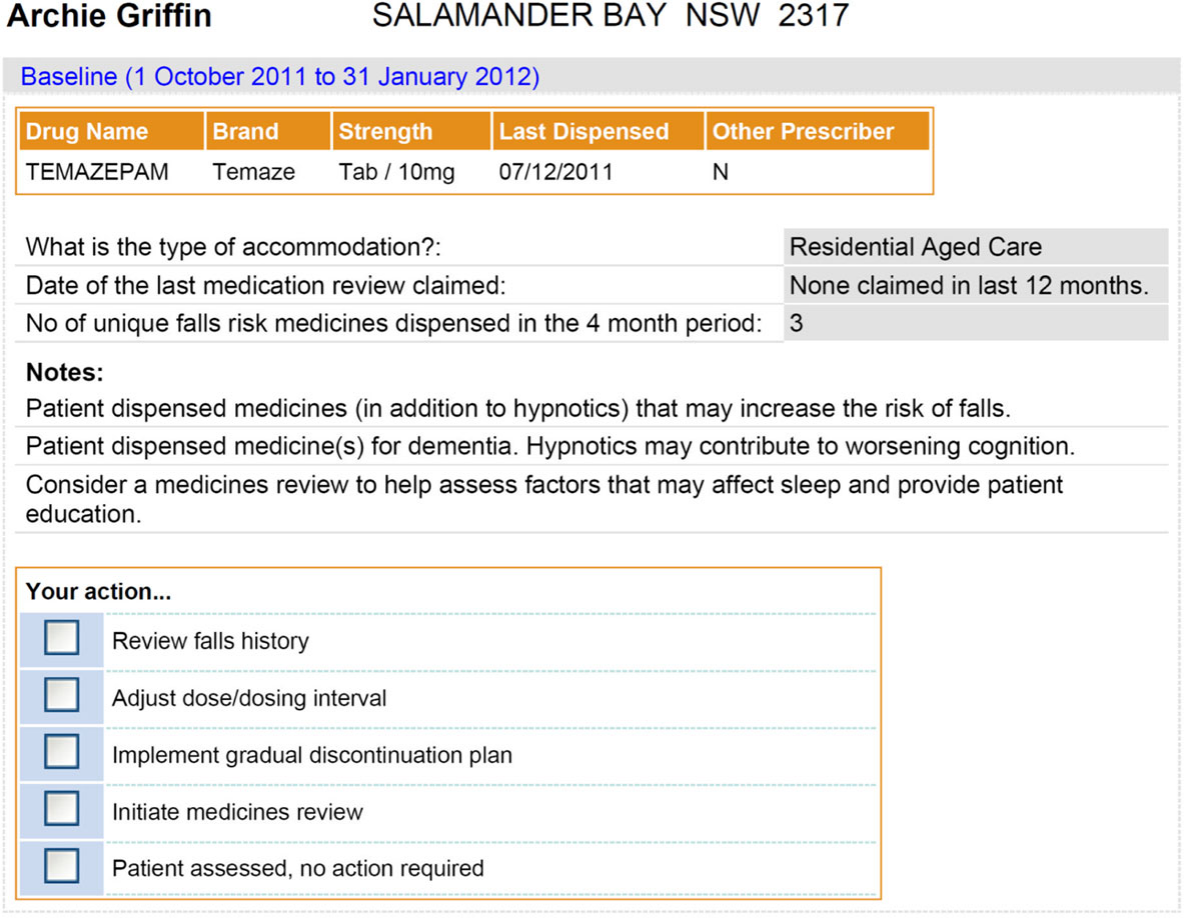
Example of patient-specific prescriber feedback mailed to doctors targeted in the Veterans’ MATES program

In both interventions, administrative health claims data were used to identify patients who were dispensed hypnotics and the data were subsequently used to create patient-specific feedback for these patients. The GPs providing care for identified veterans taking hypnotics were mailed patient-specific prescriber feedback that lists the patient’s relevant medicines, notes identifying potential problems, prompts for review (notes) and calls to action (your action) as a tool to support cognitive engagement with the materials (Fig. 2). The prompts for actions were included on the feedback for each patient, providing repetition and reinforcement of key messages, two key processes identified within social cognitive theory to support individual behaviour change (see Fig. 1) [32]. In both interventions a tailored educational brochure was enclosed to further support behaviour change by raising doctors’ awareness and knowledge of the issues [32]. The brochure explained the efficacy and risks of hypnotics in the elderly, identified the need to reduce or discontinue hypnotics to avoid adverse events, and encouraged use non-pharmacological options for managing insomnia, as well as provided a stepped guide on how to cease use to support the targeted behaviour change. As motivation is another factor identified in theoretical frameworks to support behaviour change [32], the brochure highlighted the adverse events that could be avoided if hypnotic use was ceased. (https://www.veteransmates.net.au/VeteransMATES/documents/module_materials/M31_TherBrief.pdf). In addition, the interventions referred GPs to resources for non-pharmacological management of insomnia, which included the recommendation that cognitive behavioural therapy is best delivered by a psychologist. The Department of Veterans’ Affairs subsidises access to psychologists for veterans and GPs were able to refer their patients for this service.

Consistent with the health promotion framework Precede-Proceed [33], we also targeted all other relevant stakeholders whose activities might influence hypnotic use; this included aged-care staff, pharmacists and patients themselves. At the same time as the GP intervention, educational information was mailed to all directors of care in residential aged care facilities, community pharmacies, and pharmacists accredited to perform home medicines reviews for people living in the community and residential medication management reviews for residents in aged care facilities. Four weeks after the intervention package had been sent to the health professionals, veterans meeting the eligibility criteria were mailed a consumer-focused educational brochure outlining the “myths” and “facts” about insomnia and the benefits of non-pharmacological options in the long-term. (https://www.veteransmates.net.au/VeteransMATES/documents/module_materials/M31_VetBrochure.pdf). The materials asked veterans to “make an appointment to talk to your doctor if you wish to learn more about medicine-free treatment options”. These tailored educational materials were developed by a medical writer in consultation with a clinical reference group. Prior to publication, the materials were externally peer-reviewed and endorsed by a national representative editorial committee. A national call centre provides post-implementation support services.

### Stage 2: Evaluation of interventions

Stakeholder perceptions of the service were evaluated using one-page reply forms mailed at the time of the intervention. The response forms also included commitment questions, asking doctors how many patients they would review and asking veterans if they would make an appointment with their doctor to discuss non-drug options for insomnia. Commitment questions were included in the response forms as the theory suggests positive responses are likely to further support behaviour change [34].

DVA’s prescription claims between 1 March 2007 and 31 May 2014 were used to determine the monthly hypnotic use among the veterans. This provided sufficient data points to assess trends in hypnotic use before the first intervention in March 2009 (12 months prior) and after the second intervention in June 2012 (12 months after) [35].

The primary endpoint of the study was rate of hypnotic use per month, defined as the number of veterans receiving any hypnotic in the month divided by the total number of veterans alive in that month.

Interrupted time-series and segmented regression modelling were used to determine the effect of the two interventions on the rate of hypnotics [35]. It was assumed that the rate of hypnotics was linear over the whole study period. Two breakpoints were placed at March 2009 and June 2012, and the model included terms to estimate (a) the baseline trend, defined as the average monthly change in the rate of hypnotics before the first intervention in March 2009, (b) defined as the jump or drop in the rate of hypnotic use immediately after an intervention; and (c) the average monthly change in the 12 months after each intervention, compared with the monthly trend before the intervention. To calculate the change in the slope in the 12 months after the first intervention an additional breakpoint was included in the model at March 2010. Without the additional breakpoint, 39 instead of 12 months would be used to calculate the change in slope after the first intervention. The additional breakpoint was included as we did not expect the first intervention to have an impact on the rate of hypnotics beyond 12 months. Nonstationarity, seasonality and any autocorrelation were controlled for in the time series. Stepwise backward elimination was used to determine the model of best fit. Data were analysed using SAS version 9.4 (SAS Institute Inc., Cary, NC, USA).

### Stage 3: Estimating the health consequences avoided

The number of patient-months of treatment with hypnotics avoided was calculated every month as the difference between number of hypnotics used following intervention and predicted number of hypnotics used if without intervention. The cumulative patient-months of treatment with hypnotics avoided was calculated as a sum of the number of patient-months of treatment avoided from April 2009 to May 2014.

Results from two retrospective cohort studies we conducted in the Australian veteran population were used to evaluate the health consequences of the interventions [15, 18]. These studies assessed rates of hospitalisation for confusion or falls based on the number of hypnotic or sedative medicines used, and adjusted rates are shown in Table 1.

**Table 1:** Number of hospitalisations for confusion or falls avoided assuming cessation of one hypnotic

Hospitalisation for confusion (18)							
Number of hypnotics	Number of Confusion	Person-Years	Adjusted rate per 10 years (95% CI)	Incidence rate ratio (95%)	Adjusted rate difference per 10 years	Adjusted rate difference per month	Total hospitalisations for confusion avoided
0	156	21,491	0.049 (0.032–0.074)	1.00 (1.00–1.00)	–	–	–
1	118	16,773	0.052 (0.034–0.081)	1.07 (0.84–1.36)	0.0035	0.000029	1
2	76	5078	0.12 (0.074–0.18)	2.39 (1.79–3.19)	0.064	0.00054	11
3	24	1503	0.14 (0.078–0.24)	2.79 (1.78–4.38)	0.020	0.00016	4
4	10	454	0.21 (0.10–0.43)	4.26 (2.20–8.25)	0.072	0.00060	12
> = 5	17	200	0.94 (0.51–1.73)	19.35 (11.10–33.72)	0.733	0.0061	128
Hospitalisation for falls (15)							
Number of hypnotics	Number of Falls	Person-Years	Adjusted rate per 10 years (95% CI)	Incidence rate ratio (95%)	Adjusted rate difference per 10 years	Adjusted rate difference per month	Total hospitalisations for falls avoided
0	518	20,448	0.17 (0.13–0.23)	1.00 (1.00–1.00)	–	–	–
1	527	16,945	0.21 (0.16–0.28)	1.22 (1.08–1.38)	0.038	0.00031	7
2	246	5231	0.29 (0.22–0.40)	1.70 (1.45–1.99)	0.084	0.00070	15
3 to 4	108	1946	0.34 (0.24–0.48)	1.96 (1.58–2.43)	0.045	0.00037	8
> = 5	16	189	0.55 (0.31–0.96)	3.15 (1.90–5.23)	0.21	0.0017	36

Data on number of confusion, number of falls, person-years, adjusted rates per 10 years and incidence rate ratio were taken from the two retrospective cohort studies which have been published previously (15,18)

Total number of hospitalisation for confusion or hospitalisation for falls prevented were calculated by multiplying the number of patient-months of treatment avoided, which was 20,850, with the adjusted risk difference per month

Note: We assumed cessation of one hypnotic (e.g. from two hypnotics to one) as a conservative estimate. The number of hospitalisation for confusion or hospitalisation for falls prevented would have been higher if patients had reduction of two or more hypnotics

Based on the adjusted rates reported in the two retrospective cohort studies, the adjusted rate difference between each incremental number of hypnotics and sedatives used (e.g. between zero and one hypnotic, between one and two hypnotics) was calculated. This adjusted rate difference was then multiplied by the cumulative number of patient-months of treatment with hypnotics and sedatives avoided to estimate changes in health consequences. For example, the adjusted rate difference per month of hospitalisation for confusion when discontinuing one hypnotic such as from one to zero, two to one, or three to two were 0.000029, 0.00054 and 0.00016 respectively [15] (Table 1). The total number of hospital admissions for confusion which were avoided as a result of the interventions was calculated by multiplying the adjusted rate difference by the total number of patient-months of treatment avoided. We assumed cessation of one hypnotic as a conservative estimate.

### Ethics approval

This study was approved by the University of South Australia Human Research Ethics Committee and DVA’s Human Research Ethics Committee.

## Results

### Stage 1: Implementation of national interventions

A total of 52,869 veterans were targeted in the first intervention and 21,329 veterans were targeted in the second intervention (Table 2). Of these 65.5% were also targeted in the first intervention. In the first intervention, 49.2% were men and 84.6% were living in the community while in the second intervention, 45% were men and 78% were living in the community. A total of 22,428 GPs, 15,909 pharmacists and 5327 directors of care in residential aged care facilities were targeted in both interventions (Table 2).

**Table 2:** Target audience for the Veterans’ MATES interventions to reduce use of hypnotics among Australian veterans

Date intervention commenced	Number of health professionals targeted			Criteria used to select targeted veterans	Number of veterans targeted
	GPs	Pharmacists	Directors of nursing in residential aged care facilities		
March 2009	13,203	7623	2722	Veterans dispensed at least one hypnotic between 1 January and 31 December 2008	52,869
June 2012	9225	8286	2605	Veterans dispensed at least two hypnotics between 1 October 2011 and 31 January 2012	21,329

### Stage 2: Evaluation of interventions

**Fig. 3:**
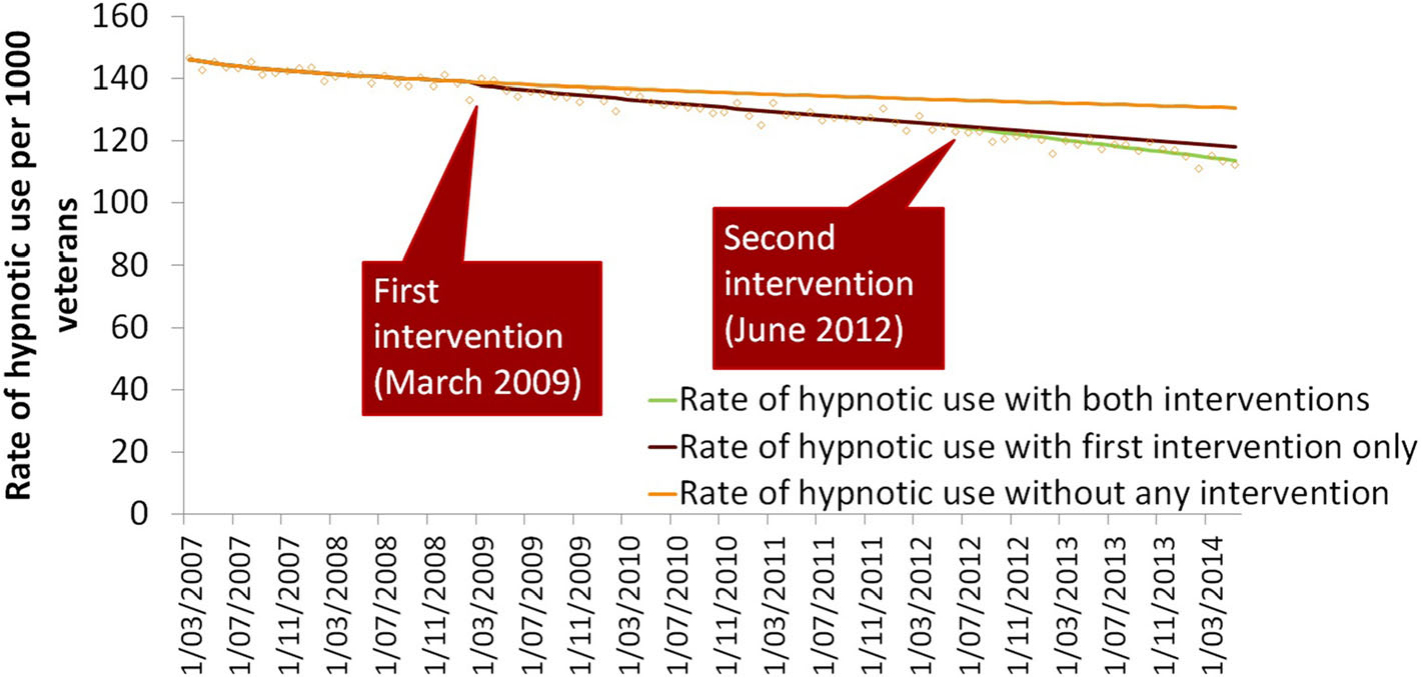
Monthly time series showing the effect of interventions on hypnotic use among Australian veterans

Hypnotic use was decreasing at the start of the study period (Fig. 3). In March 2007, prior to the intervention, 14.7% (*n* = 47,727) veterans received hypnotics, with use declining on average by 0.3% per month until February 2009 where use was 13.9% of the veteran population. After the first Veterans’ MATES intervention in March 2009 use declined significantly by a further 0.2% each month, when compared to the baseline trend (*p* = 0.006). One year after the first intervention, in March 2010, the intervention effect had attenuated and use of hypnotics was increasing by 0.2% per month. Following the second intervention in June 2012, the monthly trend in the rate of use of hypnotics was once again declining, by 0.18% each month over the 12 months of follow up (*p* = 0.049) (Fig. 3, Table 3). In March 2013, 12.1% (*n* = 27,646) veterans received hypnotics (Fig. 3).

**Table 3:** Estimates of changes in hypnotics use following each Veterans’ MATES intervention

Parameter	Estimate (95% CI)	*p*-value
Change in trend before March 2009	−0.30 (−0.35, −0.24)	<.0001
Change in level at March 2009	0.73 (−0.80, 2.25)	0.346
Change in trend after March 2009	−0.23 (− 0.39, − 0.07)	0.006
Change in level at March 2010	1.02 (− 0.25, 2.29)	0.114
Change in trend after March 2010	0.21 (0.04, 0.37)	0.0147
Change in level at June 2012	−0.41 (−1.89, 1.06)	0.579
Change in trend after June 2012	−0.18 (− 0.36, − 0.001)	0.0492

We estimate that associated with the interventions, on average, there were 500 fewer veterans using hypnotics per month. This equates to a cumulative 12,000 fewer veterans over the 24-month intervention periods who would otherwise be on hypnotics.

### Stage 3: Estimating the health consequences

The cumulative effect of both interventions from March 2009 to May 2013 resulted in a reduction of 20,850 patient-months of treatment with hypnotics. The number of hospitalisations avoided, assuming cessation of one hypnotic, is shown in Table 1. At a minimum, the interventions results in one less hospital admission for confusion and 7 less hospital admissions due to falls. If the patients were on two sedative medicines and ceased one medicine, it is estimated that this would lead to 11 fewer hospital admissions for acute confusion and 15 fewer hospital admissions for falls.

## Discussion

Our evaluation showed that the Veterans’ MATES interventions using patient-specific prescriber feedback, combined with educational information to other health professionals and consumer-focused educational brochures targeting veterans were effective in reducing hypnotic use among Australian veterans. At the start of the study in March 2007, 15% of older Australians were using hypnotics prior to the Veterans’ MATES interventions. Use reduced to 12% of the veteran population in May 2013 following both interventions. While the absolute effect size is small, the absolute effects do equate to health improvements which at a minimum were one less hospital admission for confusion and 7 less hospital admissions due to falls, as well as potential unmeasured improvements in milder forms of cognitive impairment and less serious falls.

An important lesson learned from conducting the Veterans’ MATES program is that interventions with very specific messages (e.g. reduce hypnotic use) were more effective than that interventions with more generic adverse event messages (e.g. reduce potentially inappropriate medicine use in elderly) [27]. In addition, repeated messages over time are needed to sustain improvement in use of medicines. Interventions need to be repeated to increase the persuasiveness of the messages [36]. Building on the success of the first intervention, the second intervention repeated the key messages and informed GPs of their patients who were still using hypnotics via the patient-specific prescriber feedback. The combined effect elicited sustained practice change with a further reduction in hypnotic use following the second intervention.

A successful national level intervention should always be underpinned by behavioural theories. The success of the Veterans’ MATES interventions is underpinned by the social cognitive theory [32] and the Precede-Procede health promotion framework [33], the latter which emphasises strong stakeholder engagement. During both interventions, targeted GPs were mailed patient-specific prescriber feedback, which comprised patient’s relevant medicines, a prompt for GP review (notes identifying potential problems) and calls to action as a tool to support cognitive engagement with the materials. The patient-specific prescriber feedback helped GPs to easily identify patients using hypnotics who were at increased risk of adverse events. In addition, tailored educational brochures with recommendations on management techniques and advice to patients were provided to assist GPs in resolving the potential medication-related problems. Veterans were mailed a consumer-focused educational brochure explaining the benefits of non-pharmacological options in the long-term. Engagement of other relevant stakeholders including pharmacists and directors of nursing in residential aged care facilities at a national level maximised the efficiency of outreach to support behaviour and practice change.

Mismatch between GPs’ and patients’ perceptions or expectations often hinders effective management of insomnia [26]. Patients often try to resolve sleep problems themselves by using various unproven alternative treatments which are ultimately ineffective [37]. By the time they consult GPs, patients may have the same notion that behavioural and cognitive approaches recommended by GPs will not be effective [37]. Patients may also feel that GPs cannot help them with insomnia as they think that GPs have other priorities [26]. Thus, during both interventions, veterans using hypnotics were mailed educational materials emphasizing that their GPs can recommend effective non-drug treatments and asking the veterans to make an appointment with their GPs. In contrast, GPs often assume that patients expect a hypnotic [26], or that patients already on hypnotics are resistant to stopping the hypnotics [37]. Based on veterans’ survey response in the first intervention, the second intervention highlighted to the GPs the veterans’ willingness to try non-pharmacological interventions and, for patients already taking hypnotics, their willingness to reduce the amount of hypnotics they were using.

A 2013 systematic review which included eight qualitative studies on GPs experience, one which was conducted in Australia [38], revealed that the decision to prescribe or withdraw benzodiazepines in primary care patients can be complex, uncomfortable and demanding due to reasons including time-constraints, their perception of patient expectations and the doctor-patient relationship [39]. The wish to maintain a good doctor-patient relationship and to remain competitive in the healthcare market often involve succumbing to patients’ demand for medicines [40, 41], which in this context meant prescribing hypnotics. As patients may often have attempted many different, ineffective, alternatives prior to consulting GPs [37], many would expect a prescription for hypnotics by the time they consult their GPs. To address these issues, patient empowerment and involvement in withdrawing hypnotics was accounted for in constructing the framework of our intervention. The tailored educational brochures sent to veterans described the non-drug options for insomnia, the harms of hypnotic use and the potential to stop hypnotics. A 2017 Cochrane systematic review reported that use of decision aids such as brochures is effective in improving patients’ knowledge of treatment options, patient-clinician communication, and patients’ ability to participate in shared decision-making [42]. The review also showed that patients were more likely to choose the more conservative treatment option when they were better informed about the benefits and harms of treatment [42]. It was anticipated that increasing patient understanding and awareness on the risks of hypnotics would make it easier for GPs to communicate the rationale for stopping hypnotics.

Older people are more vulnerable to the side effects of hypnotics due to multimorbidity and multiple medicines that they are taking [29, 30]. Insomnia management using hypnotics becomes particularly problematic when concomitant medicines not indicated for insomnia treatment also have sedative properties. Many studies have attempted to evaluate the feasibility of withdrawing or reducing hypnotic use in older people and the subsequent improvements in clinical outcomes [25, 43]. A 2017 systematic review of randomised controlled trials and non-randomised studies evaluated interventions to deprescribe benzodiazepines and benzodiazepine related drugs (Z-drugs) in adults aged 65 years and above [25]. The included studies were published between January 1995 and July 2015 and involved between 14 and 259 patients aged 65 years and above. One study included a GP-targeted intervention while other studies used pharmacological substitution with melatonin, or mixed approaches such as patient education with tapering advice or temporary pharmacological substitution with psychological support. Different interventions had variable discontinuation rates of between 27 and 80%. Sustainability of these interventions was unknown due to the short follow-up ranging from six weeks to one year. If measured, these intervention trials reported improvements in clinical outcomes using surrogate endpoints such as questionnaire scores, hand grip strength and balance [25, 43], without reporting important and clinically relevant health consequences such as hospitalisations for falls or confusion. While randomised and non-randomised trials to withdraw or reduce use of hypnotics showed promising results, such trials are expensive, resource-intensive and time-consuming to conduct. Furthermore, these studies were able to target only a small number of patients. The small sample sizes and short follow-up also meant the studies lacked the capacity to detect relatively less common but clinically relevant outcomes such as hospitalisations for falls or confusion. In contrast, our study assessed the effectiveness of reducing hypnotic use among older people at a national level. Our results showed that the interventions achieved sustained reduction in hypnotic use, as demonstrated by the monthly decrease rate in hypnotic use which persisted up to 12 months after each intervention.

An important implication of the Veteran’s MATES programme is the health and economic consequences associated with the reduction in hypnotic use following both interventions. We have previously demonstrated that hypnotic use was associated with increased risk of hospitalisation for falls [15], and hospitalisation for acute confusion [18] in the same population. A previous meta-analysis has also shown an increased risk of fractures with hypnotic use [44]. Using the risk estimates calculated using the Australian veterans’ data [15, 18], the same population that was targeted by our intervention, we were able to estimate the impact of reduction in hypnotic use on clinically important patient health outcomes. The cumulative reduction in patient-months of hypnotic use was estimated to lead to a reduction in the number of hospitalisations for falls and hospitalisations for confusion. It is important to remember that the health consequences that we have estimated are likely to be an underestimate, as many people have falls or experience confusion that does not result in hospital admission, but which may still have a substantial impact on their health or quality of life. The costs associated with conducting the program are offset by the cost-savings associated with improved use of medicines and associated improvements in health outcomes for veterans, arising from the Veterans’ MATES program.

Our study has several strengths. We used data from a large sample of older Australians and tracked real-time use of hypnotics at the national level. Our results are likely to be generalisable to all older Australians as they had similar rates of medicine use and medical services when compared with veterans targeted in this intervention [45, 46]. While the results on impact of cumulative reduction in hypnotic use on health outcomes should be regarded as an estimate, it is likely a conservative estimate of improvements in health outcomes.

One of the limitations of this study was that we do not know the number of targeted stakeholders who actually read the information. We only had information on the number of letters returned to sender, which was below 1%. We were unable to evaluate the usefulness of tailored educational brochures in influencing veterans’ willingness to stop hypnotic use or initiate conversations with GPs to withdraw their hypnotics. Similarly, we had no information on the extent to which pharmacists and directors of nursing in residential aged care facilities affected trends in hypnotic use, although the intervention targeted these groups to assist with reinforcement of key messages. Measuring changes in GP prescribing as a result of our intervention is difficult. This is because veterans in Australia are able to visit any GP and do not always visit the same GP. This means that the medicines that they are taking may be prescribed by different GPs. We were unable to determine how many veterans were referred to psychologists to receive cognitive behavioural therapy, and whether there were enough psychologists available to provide cognitive behavioural therapy for the patients referred to them in a timely manner. Patients awaiting cognitive behavioural therapy from a qualified psychologist are unlikely to have been able to reduce their use of hypnotics while waiting for this service and this may have influenced the results of our study.

## Conclusions

The Veterans’ MATES interventions were effective in reducing hypnotic use among older Australians. The monthly decreasing trend was sustained at 12 months after intervention. The cumulative number of patient-months of hypnotic treatment avoided was estimated to accrue health benefits with a reduction in hospital admissions due to falls and hospital admissions for acute confusion. Our results show that a theoretically based, national intervention with follow-up to reduce use of potentially harmful medicines targeting multiple stakeholders and involving repetition of specific key messages can achieve sustained practice change.

## Abbreviations

ATC: Anatomical, Therapeutic and Chemical Classification
BEACH: Bettering the Evaluation and Care of Health
DVA: Department of Veterans’ Affairs
GPs: general practitioners
MATES: Medicines Advice and Therapeutics Education Services
NAMCS: National Ambulatory Medical Care Survey
